# Supplementary material for: Integrating genomic structural equation modeling and experimental validation to unravel the genetic basis of male genital lichen sclerosus
Source: Front Immunol. 2026 Jul 16;17:1857945. doi: 10.3389/fimmu.2026.1857945 (PMC13435250; doi:10.3389/fimmu.2026.1857945)
Supplement: Supplementary file 6 [file SupplementaryFile5.zip › result/fuma_PSYCH_factor_sCCA1_focus.chr1.chr1.25516845.27401867.pop1.pdf]

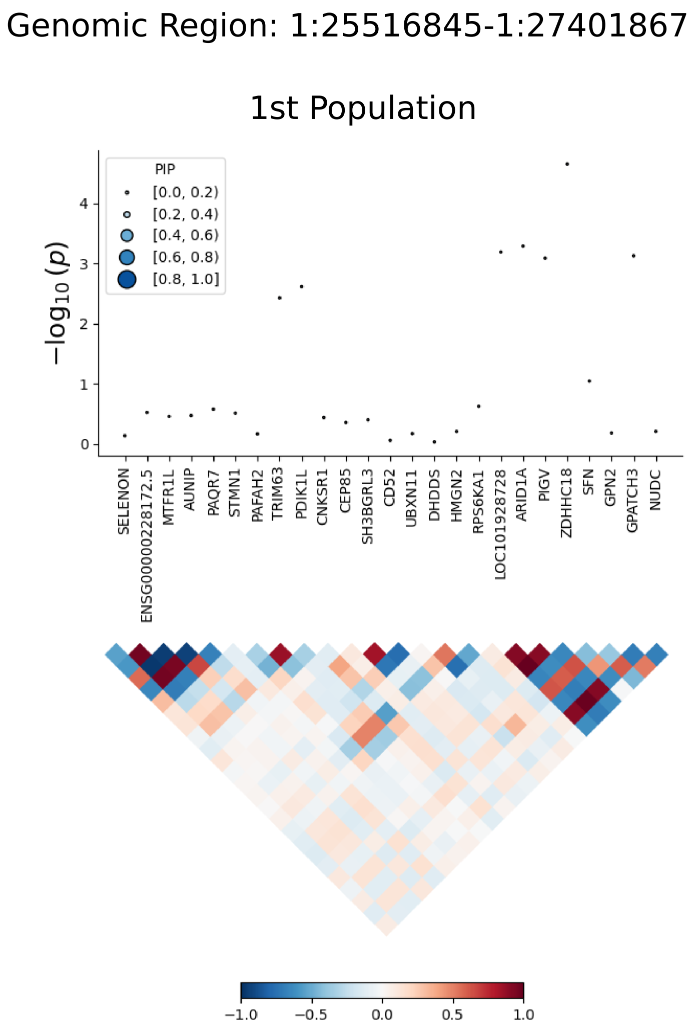

Genomic Region: 1:25516845-1:27401867
1st Population
PIP
[0.0, 0.2)
[0.2, 0.4)
[0.4, 0.6)
[0.6, 0.8)
[0.8, 1.0]
$-\log_{10}(p)$
0
1
2
3
4
SELENON
ENSG00000228172.5
MTFR1L
AUNIP
PAQR7
STMN1
PAFAH2
TRIM63
PDIK1L
CNKSR1
CEP85
SH3BGRL3
CD52
UBXN11
DHDDS
HMGN2
RPS6KA1
LOC101928728
ARID1A
PIGV
ZDHHC18
SFN
GPN2
GPATCH3
NUDC
−1.0
−0.5
0.0
0.5
1.0
